# Next generation sequencing reveals widespread trypanosome diversity and polyparasitism in marsupials from Western Australia

**DOI:** 10.1016/j.ijppaw.2018.01.005

**Published:** 2018-01-28

**Authors:** Crystal Cooper, Sarah Keatley, Amy Northover, Alex W. Gofton, Frances Brigg, Alan J. Lymbery, Louise Pallant, Peta L. Clode, R.C. Andrew Thompson

**Affiliations:** a Centre for Microscopy, Characterisation and Analysis, University of Western Australia, Stirling HWY, Crawley, WA 6009, Australia; b School of Veterinary and Life Sciences, Murdoch University, 90 South Street, Murdoch, WA 6150, Australia; c State Agriculture and Biotechnology Institute, School of Veterinary and Life Sciences, Murdoch University, WA 6150, Australia; d Fish Health Unit, School of Veterinary and Life Sciences, Murdoch University, South Street, Murdoch, WA 6150, Australia

**Keywords:** *Trypanosoma* spp., *Bettongia penicillata*, *Trichosurus vulpecula*, Host-parasite relationship, 18S rDNA targeted amplicon sequencing

## Abstract

In Western Australia a number of indigenous *Trypanosoma* spp. infect susceptible native marsupials, such as the woylie (*Bettongia penicillata*), brushtail possum (*Trichosurus vulpecula*), and chuditch *(Dasyurus geoffroii*). Two genotypes of *Trypanosoma copemani* (identified as G1 and G2) have been found in the woylie, and G2 has been implicated in the decline of this host species, making its presence of particular interest. Here we used targeted amplicon next generation sequencing (NGS) of the *Trypanosoma* 18S rDNA loci on 70 *Trypanosoma*-positive marsupial blood samples, to identify *T. copemani* genotypes and multiple *Trypanosoma* infections (polyparasitism) in woylies and cohabiting species in Western Australia. Polyparasitism with *Trypanosoma* spp. was found in 50% of the wildlife sampled, and within species diversity was high, with 85 zero-radius operational taxonomic units (ZOTUs) identified in nine putative parasite species. *Trypanosoma copemani* was assigned 17 ZOTUs and was identified in 80% of samples. The most abundant ZOTU isolated (63%) differed slightly from the published genotype of G1, and G2 was the second most abundant ZOTU (14%). Trypanosome diversity was significantly greater in woylies than in brushtail possums, and parasite community composition also differed significantly between these host species. One novel *Trypanosoma* spp. genotype (*Trypanosoma* sp. ANU2) was found in 20% of samples. A species of *Crithidia* was detected in a woylie, and two avian trypanosomes (*Trypanosoma avium* and *Trypanosoma* sp. AAT) were identified in woylies for the first time.

## Highlights

• Extensive trypanosome diversity and polyparasitism in south Western Australia.
• A significant difference between trypanosomes infecting woylies and brushtail possums.
• Evidence that the current species-level taxonomy of Australian trypanosomes may need revision.

## Graphical abstract

**Figure:**
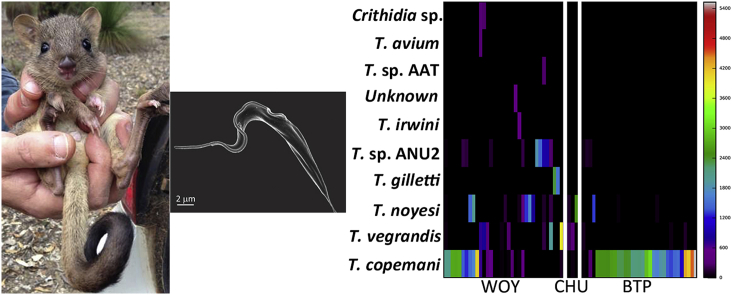

## 1 Introduction

Since Europeans arrived in Australia over 200 years ago, 30 native mammals have become extinct, with 30% of the surviving terrestrial mammalian population (excluding bats), threatened with extinction (Woinarski et al., 2015). The woylie or brush-tailed bettong, (*Bettongia penicillata*) is one of Australia's most critically endangered marsupials (Wayne et al., 2008). Once occupying a substantial portion of southern Australia, the woylie is now confined to just three regions in the south west of Western Australia (WA) after experiencing a 90% population decline over seven years (Yeatman and Groom, 2012, Wayne et al., 2013). Two of the remaining three wild populations occur in the Upper Warren Region (UWR) in south west WA (Kingston and Perup) (Yeatman and Groom, 2012). In addition to direct human impact, there is also evidence to suggest that pathogens have played a role in population declines, particularly at the local level (Thompson et al., 2009, Thompson et al., 2010), with remaining woylie populations parasitised by indigenous *Trypanosoma* spp. (Botero et al., 2013, Thompson et al., 2013a, Thompson et al., 2013b). Other susceptible marsupials such as the brushtail possum (*Trichosurus vulpecula*) and the near-threatened chuditch (*Dasyurus geoffroii*) also share habitats with woylies and are susceptible to *Trypanosoma* spp. infection.

One species of *Trypanosoma* implicated in the woylie decline is *Trypanosoma copemani*, which is an indigenous Australian trypanosome that has been isolated from a number of marsupials. It has two unique isolates, which have been isolated from the woylie - *T. copemani* genotype 1 (G1) and *T. copemani* genotype 2 (G2) (Botero et al., 2013). While G1 is closely related to other genotypes found in marsupial species across Australia (Noyes et al., 1999, Hamilton et al., 2005, Austen et al., 2009, Botero et al., 2013), G2 is less common and genetically divergent from other *T. copemani* genotypes at the 18S rDNA and GAPDH loci (Botero et al., 2013). Additionally, *T. copemani* G2 has been found to have a higher prevalence in declining populations than in stable populations of the woylie (Botero et al., 2013, Thompson et al., 2013b). Furthermore, G2 has been detected in woylie tissues via PCR, observed in histological sections of woylie heart tissue, isolated in culture from woylie blood samples, and observed invading mammalian cells *in vitro* (Botero et al., 2013, Botero et al., 2016). Intracellular behaviour is unusual in *Trypanosoma* spp. (Cooper et al., 2016), but is responsible for the chronic pathogenicity of *T. cruzi* (the causative agent of human Chagas disease), which leads to an incurable infection and significant mortality in humans (Hoare, 1972).

Current generic PCR assays can readily detect *Trypanosoma* spp. infection in wildlife blood samples to the species level of currently described species but it cannot differentiate between genotypes of the same species due to the small number of nucleotide polymorphisms separating them (Botero et al., 2013). As a result, Sanger sequencing (Sanger et al., 1977) is commonly used to genotype samples, which can be costly and time consuming. Additionally, polyparasitism with different indigenous *Trypanosoma* spp. has been reported in Australian wildlife between *T. copemani*, *T. vegrandis*, and *T. noyesi* by sampling different tissues from the same host or by using species specific PCR primers without Sanger sequencing (Botero et al., 2013, Thompson et al., 2013b). This indicates that the abundance and diversity of *Trypanosoma* spp. is often misrepresented without using both direct Sanger sequencing and targeted primers for all species present, and that would be additionally costly and time consuming.

Recent advances in next-generation sequencing (NGS) technologies allow for new approaches to investigate the population genetics and ecology of microparasites and free-living microorganisms (Van Dijk et al., 2014). Next-generation sequencing enables the parallelisation of millions of sequencing reactions, making it feasible to investigate species diversity and prevalence in large populations. For example, targeted amplicon sequencing of bacterial 16S rRNA gene regions is commonly used to profile the bacterial communities that inhabit a variety of organisms including humans (Kuczynski et al., 2010), arthropods (Gofton et al., 2015), and soil (Bartram et al., 2011). High throughput sequencing methods have also been applied to 18S rDNA regions for eukaryotic parasites such as *Plasmodium* spp. (Carlton et al., 2013, Bunnik et al., 2013), *Cryptosporidium* spp. (Paparini et al., 2015), *Eimeria* spp. (Vermeulen et al., 2016), and recently for the first time *Trypanosoma* spp. (Barbosa et al., 2017). This method is particularly valuable in that different species can be identified from relatively short (300–500 bp) targeted gene regions, which is common in *Trypanosoma* spp. (Noyes et al., 1999, Austen et al., 2009, Averis et al., 2009, Botero et al., 2013, Thompson et al., 2013b, Barbosa et al., 2017).

The aim of the current study was to investigate the diversity of trypanosomes infecting the woylie and cohabiting species (chuditch and brushtail possum) within the UWR of Western Australia. We were particularly interested in *T. copemani* genotypes, because of the perceived risk posed by *T. copemani* G2 to the remaining woylie populations.

## 2 Materials and methods

### 2.1 Sample collection

Blood samples were collected from woylies, brushtail possums, and chuditch in the UWR throughout a three year project between Murdoch University (MU) and the Western Australian Department of Parks and Wildlife (DPaW), under MU animal ethics permits WC2350-10 and RW2659/14, and DPaW animal ethics permit SF010420. During the project, woylies were translocated from Perup Sanctuary in the UWR, an enclosed reserve, into two wild destination sites: Warrup East and Walcott (Northover et al., 2015). Sampling was carried out on both resident marsupials and translocated woylies before and after woylie translocation. Animals were captured using Sheffield traps (Sheffield Wire Products, Welshpool, WA) set at sunset that were baited with sardines, peanut butter and oats. During processing a total of 600 μl of blood was collected in an EDTA tube from either the lateral caudal vein (woylie, performed conscious) and/or the jugular vein (brushtail possum and chuditch, under anaesthesia). Blood smears were air dried and stained with DiffQuik before remaining blood was stored at −20 °C for molecular analyses. After sampling, animals were released into their destination site at sunrise. A total of 70 *Trypanosoma*-infected blood samples were selected from this study for NGS (Table 1), based on previous PCR and Sanger sequencing results that indicated trypanosome infection (Northover et al.; unpublished data).

**Table 1:** Marsupial blood samples used in targeted amplicon next generation sequencing (NGS). Sample ID of collected samples and individual animals that are duplicated in the dataset because they were sampled on different dates (Dup) are included. Host species (WOY = woylie, BTP = brushtail possum, CHU = chuditch), site location (PER = Perup, WAR = Warrup East, WAL = Walcott), before (pre) or after (post) translocation, *Trypanosoma* that were identified in NGS (C = *Trypanosoma copemani*, V = *T. vegrandis*, N = *T. noyesi*, A = *T.* sp. ANU2, G = *T. gilletti*, I = *T. irwini*, AT = *T.* sp. AAT, AA = *T. avium*, U = unknown, CR = *Crithidia* spp.), and the ZOTUs that infected each sample are also included.

Sample ID	Dup	Host	Site	Trans.	Trypanosoma	ZOTUs
4466_S34		BTP	PER	Pre	C	1
4762_S8		WOY	PER	Pre	C,A	2,4,47,79
4771_S35	D1	BTP	PER	Pre	C	1
7904_S18	D1	BTP	PER	Pos	C	1
4772_S79		BTP	PER	Pre	C	1
4827_S5		BTP	PER	Pre	C	1
4829_S43		WOY	PER	Pre	C	1
4832_S36	D2	BTP	PER	Pre	C	1
7912_S72	D2	BTP	PER	Pos	C	1
4835_S21		WOY	PER	Pre	N,C	3,14,51,78,40,44,42
4838_S83		BTP	PER	Pre	C	1
4853_S86		WOY	WAR	Pre	C,N,A,V	1,2,3,4,6,
4865_S2		BTP	WAL	Pre	C	1
4871_S76		CHU	WAL	Pre	V	7,50,6,46
4944_S62		BTP	WAL	Pre	C	1
4953_S67		BTP	WAL	Pre	C	1
4976_S96		BTP	WAL	Pre	C,N	1,3
4977_S3		WOY	WAL	Pre	G	9
4986_S78	D3	BTP	WAL	Pre	C	1
6986_S15	D3	CHU	WAR	Pos	C,N,A	1,3,63,67,64
4990_S58		WOY	WAL	Pre	C	1
4995_S74		BTP	WAR	Pre	C	1
4996_S6		BTP	WAR	Pre	C,A	2,4
4998_S80	D4	BTP	WAR	Pre	A	31,59,62,66
6601_S7	D4	BTP	WAR	Pos	C	1
5001_S32		BTP	WAR	Pre	C,V	1,6,7,46
5034_S88		WOY	WAL	Pre	V	17,25,29,33,35,32
5046_S47	D5	WOY	WAL	Pre	V	5,10
7034_S1	D5	WOY	WAL	Pos	C	1,2
5068_S31	D6	BTP	WAL	Pre	C	1
7012_S23	D6	BTP	WAL	Pos	C	1
5071_S55		BTP	WAL	Pre	C	1
5072_S29		WOY	WAL	Pre	C,V	1,6
5078_S66		BTP	WAL	Pre	V	5
5080_S17		WOY	WAL	Pre	C	1
5099_S30		BTP	WAR	Pre	C,V	1,83
5127_S77		WOY	PER	Pre	C	1
5144_S85		WOY	PER	Pre	N,CR,	3,26,41,65,75,18
5147_S48		WOY	PER	Pre	C,N,AA	1,3,21
5158_S90		BTP	WAR	Pre	C,V	1,49,71,85
5245_S10		WOY	WAR	Pos	A	4,16
5266_S93		WOY	PER	Pre	A	4
5268_S11		WOY	PER	Pre	C,A	1,2,4
5323_S59		WOY	PER	Pre	C,G	1,8
5366_S24		WOY	PER	Pre	C	2
6237_S81		WOY	WAL	Pos	C	1
6251_S91		WOY	WAR	Pos	A,V,N	4,5,34,36,56,72,73,74,76, 3,4,82
6370_S28		WOY	WAL	Pos	A,V,C	4,5,12
6410_S13	D7	WOY	WAL	Pos	V	6,15,22
6709_S14	D7	WOY	WAL	Pos	N,A,AT,V,C	1,3,4,24,37,45,48,38,61,58
6415_S40		WOY	WAL	Pos	C,N	2,3,19,28,30,55
6473_S63		WOY	WAL	Pos	C,V,I,	2,5,13,56,64,77
6587_S16		BTP	WAL	Pos	C,N,A	2,3,4,53,81
6594_S27		WOY	WAL	Pos	V	6,54
6598_S75		WOY	WAL	Pos	V,U,N,C	5,20,27,52,69
6714_S52		WOY	WAL	Pos	N,C	23,39
6838_S84		BTP	WAL	Pos	C	1
6839_S82		BTP	WAL	Pos	C,V	1,43,84
6840_S71		WOY	WAL	Pos	C	1
6850_S50		BTP	WAL	Pos	C	1
6857_S19		BTP	WAL	Pos	C	1
6978_S70		BTP	WAR	Pos	C	1
6988_S54		BTP	WAR	Pos	C	1
7000_S46		BTP	WAL	Pos	C	1
7024_S41		CHU	WAL	Pos	N,V	3,5,68,70
7036_S49		WOY	WAL	Pos	C,V	1,5,43,60,11
7042_S51		WOY	WAR	Pos	C,A	2,4
7909_S60		BTP	PER	Pos	C	1
7938_S22		WOY	WAR	Pos	A	4
8068_S87		WOY	WAL	Pos	N,C	3,39,57,80

### 2.2 DNA extraction and 18S rDNA amplification

DNA was extracted from 200 μl of anticoagulated blood using the QIAamp 96 DNA blood kit (Qiagen, Hilden, Germany) following the manufacturer's instructions, and eluted in 60 μl of buffer. Samples were initially screened using generic *Trypanosoma* spp. 18S rDNA PCR primers (SLF (5′ GCTTGTTTCAAGGACTTAGC 3′), S762R (5′ GACTTTTGCTTCCTCTAATG 3′), S825F (5′ ACCGTTTCGGCTTTTGTTGG 3′), and SLIR (5′ ACATTGTAGTGCGCGTGTC 3′) in a nested PCR protocol (Maslov et al., 1996, McInnes et al., 2009), under the following conditions for the primary reaction: an initial pre-amplification step of 94 °C for 5 min, 50 °C for 2 min, and 72 °C for 4 min; followed by 35 cycles of 94 °C for 30 s, 55 °C for 30, and 72 °C for 2 min and 20 s; and a final extension step at 72 °C for 7 min. The nested reaction was performed under the following conditions: an initial pre-amplification step of 94 °C for 5 min, 50 °C for 2 min, and 72 °C for 4 min; followed by 35 cycles of 94 °C for 30 s, 56 °C for 30 s, and 72 °C for 2 min and 20 s; and a final extension step at 72 °C for 7 min. Both primary and nested reactions were performed in 25 μl volumes consisting of 1 μl of DNA, 0.8 μM of each primer, 200 μM of dNTPs, 2 mM of MgCl_2_ and 0.2 μl of Taq DNA polymerase (Thermo Fisher Scientific Inc.). *Trypanosoma*-positive samples were then screened using clade-specific primers for *T. copemani, T. vegrandis,* and *T. noyesi* (Botero et al., 2013). A known positive control (usually *T. copemani* or *T. noyesi*) was used for each PCR and negative controls were used to detect contamination of reagents and PCR products. All amplification reactions were performed in a PT100 thermocycler (MJ-Research). PCR amplicons were electrophoresed through 2% agarose gels and visualised with SYBR Safe Gel Stain (Invitrogen, USA). Single bands were produced that were excised from the gel (18S rDNA: ∼1.4 kb), purified using an in-house filter tip method (Tautz and Renz, 1983, Yang et al., 2013), and stored at −20 °C until further use.

### 2.3 NGS 18S rDNA library preparation and targeted amplicon sequencing

Targeted *Trypanosoma* 18S rDNA amplicon libraries were prepared as described by Barbosa et al. (2017). Briefly, amplicons were generated with the generic *Trypanosoma* nested PCR assay with external primers S825F and TrypAllR (5′ GACTGTAACCTCAAAGCTTTCGC 3′), and nested primers S825F and S662R (5′ GACTACAATGGTCTCTAATC 3′) (Barbosa et al., 2017). Nested primers also contained MiSeq adapter sequences at the 5′ end (Illumina Demonstrated Protocols: Metagenomic Sequencing Library Preparation). Each sample was uniquely indexed with Illumina Nextera XT indices (Illumina, San Diego, USA), and the final sequencing library was pooled and prepared according to the manufacturers recommendations (Illumina Demonstrated Protocols: Metagenomic Sequencing Library Preparation). The library was sequenced on an Illumina MiSeq using a 500 cycle v2 kit (Illumina, San Diego, USA) according to the manufacturer's recommendations to produce amplicons approximately ∼450 bp in length (250 bp paired-end reads).

### 2.4 Quality filtering and taxonomic assignment

Raw paired-end reads were merged with USEARCH v. 9.2 (Edgar, 2017) with a minimum of 50 nt overlap and no gaps allowed in the merged alignments. Only sequences that contained perfect (no mismatches) primer sequences were retained for analysis, and primer sequences and distal bases were trimmed from the sequences in Geneious v. 8.1 (Biomatters, New Zealand). Sequences were then quality filtered to allow only reads with <1% expected error as calculated with USEARCH v. 9.2 fastq_filter algorithm, and sequences >300 nt remained in the dataset. Sequences with ≤10 replicate reads were also removed from the dataset. Chimeras were removed and sequences were denoised with UNOISE2 (Edgar, 2017) into zero-radius operational taxonomic units (ZOTUs), effectively removing reads that were the result of PCR and/or sequencing error, and leaving more biologically correct sequences for taxonomic assignment. Taxonomic assignment was performed by aligning ZOTUs to a reference database of known 18S rDNA *Trypanosoma* spp. sequences downloaded from Genbank, which were aligned using MUSCLE (Edgar et al., 2004) (Supplementary Table 1). Any ZOTU that was not identical to known *Trypanosoma* spp. were investigated by comparing to the Genbank database using BLAST (Altschul et al., 1990). The phylogeny of the 85 identified ZOTUs and a sub-set of reference strains including two outgroups (*Leptomonas* spp. and *Phytomonas serpens*) downloaded from Genbank was investigated using Bayesian analysis (10,000,000 generations, sampling frequency of 1,000, and burn-in 3000), of the 377 bp rDNA sequence, was run in Mr Bayes v. 3.1.2 (Ronquist and Huelsenbeck, 2003), which confirmed species-group assignment.

### 2.5 Analyses of infracommunity diversity and composition

Following taxonomic assignment of ZOTUs, heatmaps of all samples (*n* = 70) were generated using QIIME v. 1.9.2 (Caporaso et al., 2010) to observe trypanosome abundance and diversity within individual hosts. Diversity analysis was performed on the individual animals (*n* = 56) in the dataset, excluding the same animal trapped on a different date (Table 1). ZOTU diversity within hosts was measured by the number of ZOTUs (S) and the Shannon-Wiener index (H). Differences in these parameters among host species were assessed by a non-parametric Wilcoxon sum-rank test. Similarities in ZOTU composition among hosts were estimated from square root transformed abundance data using the Bray-Curtis coefficient. Differences in ZOTU composition among host species were visualised using principle coordinate plots, implemented in QIIME v. 1.9.2 and tested for significance by a permutation procedure applied to the pairwise similarity matrix (one-way ANOSIM, implemented in PRIMER v. 6.0; Clarke and Gorley, 2006). The contribution of individual ZOTUs to differences in composition among host species was assessed by averaging the Bray-Curtis coefficients for each ZOTU over all pairwise host combinations, using the SIMPER procedure in PRIMER v. 6.0 (Clarke and Gorley, 2006).

### 2.6 Identification and phylogenetic analysis of Trypanosoma sp. ANU2

A novel *Trypanosoma* sp. genotype was identified in the woylie designated *T.* sp. ANU2 using hemi-nested PCR and Sanger sequencing (Northover et al.; unpublished data). This was subsequently identified in the brushtail possum and chuditch and further characterised in this study using NGS and submitted to Genbank under the accession number MF459652. A phylogenetic analysis was conducted to investigate its evolutionary lineage. Samples positive for *T.* sp. ANU2 were sequenced using the generic PCR primers in both directions using an ABI Prism™ Terminator Cycle Sequencing Kit on an Applied Biosystems 3730 DNA Analyser (Applied Biosystems, California, USA). A multiple-sequence alignment of 1406 bp containing the new isolate, 45 sequences downloaded from Genbank, and two outgroups (*Trypanosoma brucei gambiense* and *Trypanosoma brucei rhodesiense*), was conducted using MUSCLE. A GTR + G + I substitution model selected by jModelTest (Posada, 2008) was used in the construction of neighbour-joining, maximum-likelihood, and bayesian analysis trees. Bootstrap support for 1000 replicates was performed for neighbour-joining and maximum-likelihood analysis using MEGA v. 6 (Tamura et al., 2013), and bayesian posterior probabilities were generated using Mr Bayes v. 3.1.2 (10,000,000 generations, sampling frequency of 1,000, burn-in 3000).

## 3 Results

### 3.1 Trypanosoma spp. identified in NGS 18S rDNA targeted amplicon sequencing

**Fig. 1:**
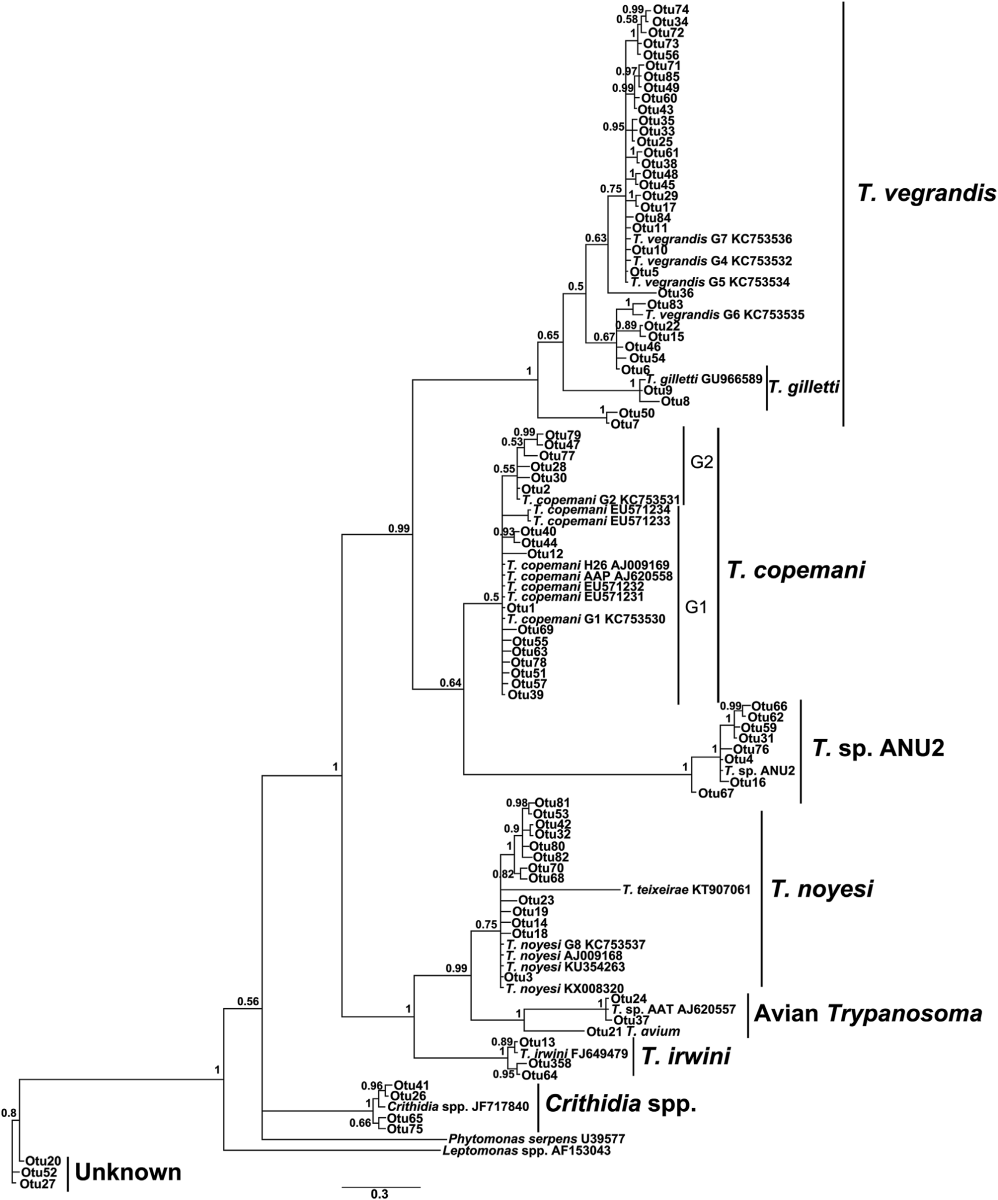
Phylogenetic relationships between *Trypanosoma* spp. ZOTUs assigned to nine species groups compared to 24 representative reference strains downloaded from Genbank. *Phytomonas serpens* and *Leptomonas* sp. were used as outgroups. Bayesian analysis was used to produce tree topology and posterior probability is shown at nodes. Scale bar represents substitution per site.

**Fig. 2:**
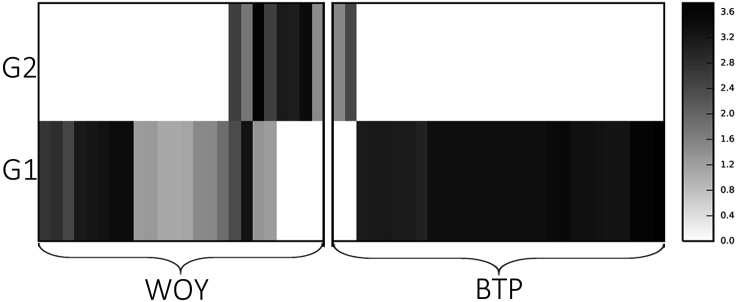
Abundance and diversity map of *Trypanosoma copemani* genotype 1 (G1) and genotype 2 (G2) positive samples. ZOTUs were sorted into G1 or G2 based on phylogenetic inference shown in rows, while columns are individual marsupial blood samples from infected individuals. The map represents samples separated by host species, which were WOY = woylie, or BTP = brushtail possum. Grayscale indicates number of sequences obtained from that sample as shown in the scale of intensity on the right (log transformed abundance).

**Supplementary Fig. 1:**
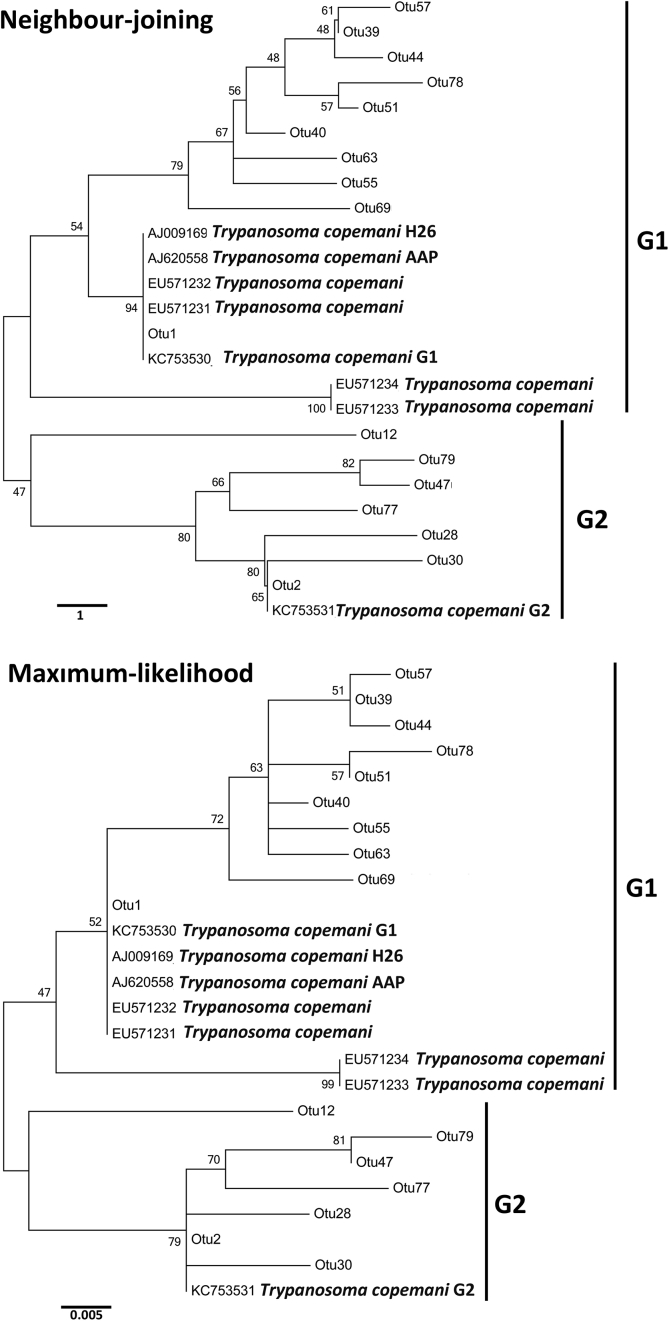
Phylogenetic assignment of *Trypanosoma copemani* ZOTUs separating ZOTUs as either *T. copemani* genotype 1 (G1) or genotype 2 (G2). Neighbour-joining and maximum-likelihood tree searches are shown and bootstrap support for 1000 replicates is shown. Scale bar represents substitution per site.

From the 70 blood samples that tested positive for trypanosomes from 56 wildlife hosts, genotypes from 85 different ZOTUs were identified, which were assigned to nine species groups (Table 1, Fig. 1). The most common trypanosome identified in the 56 blood samples was *T. copemani* with 17 unique ZOTUs in 52 individual hosts, including all three host species. The most abundant genotype isolated was most similar to G1, differing in only 2 bp (indels) and found in 44 samples from 41 hosts. The second most prevalent genotype was identical to G2 and isolated in 10 samples from 10 hosts (woylies and brushtail possums). There were 15 other ZOTUs of *T. copemani* (Fig. 1) that were assigned to either G1 or G2 based on the phylogenetic topology (Fig. 1, Supplementary Fig. 1). Brushtail possums were infected mainly with G1, while woylies were commonly infected with both G1 and G2 (Fig. 2).

Both *T. vegrandis* and *T. gilletti* were also found in all three host species, with 34 ZOTUs in 20 samples from 19 hosts, and *T. noyesi* had 13 ZOTUs in 14 samples from 14 hosts, including woylies and brushtail possums. Phylogenetic analysis was unable to separate the large number of *T. vegrandis*/*T. gilletti* ZOTUs into two separate species, which may be due to the small region of 18s rRNA amplified, and consequently these were all assigned to the same species complex (Fig. 1). *Trypanosoma irwini* was identified as three ZOTUs in one woylie sample. Oddly, known avian trypanosome sequences were also identified in two woylie samples including *T*. sp. AAT (two ZOTUs) and *T. avium* (one ZOTU) (Fig. 1).

Additional to known *Trypanosoma* spp. a *Crithidia* spp. was isolated from a woylie that did not match any known *Crithidia* spp. in Genbank (accession MF459653). The three ZOTUs from a single woylie host did not align with any known *Trypanosoma* spp. in GenBank (Fig. 1). One sample containing a unique sequence with three ZOTUs did not align with any known *Trypanosoma* spp. in Genbank, however considering its placement at the base of the phylogenetic tree it is unlikely to be a *Trypanosoma* sp. (Fig. 1).

**Fig. 3:**
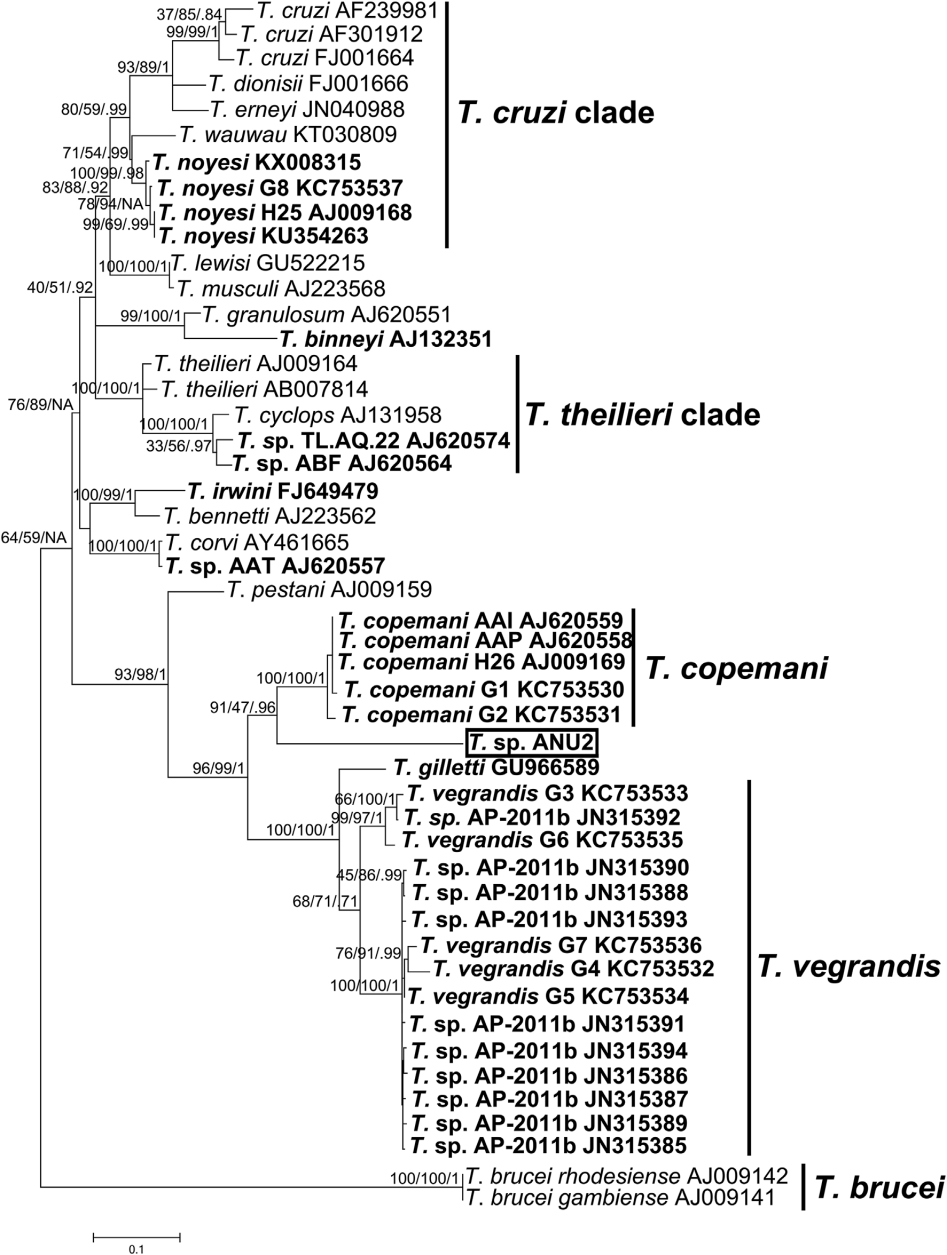
Phylogenetic relationships between *Trypanosoma* sp. ANU2 and other members of the *Trypanosoma* genus. Maximum likelihood tree is shown. Neighbour-joining and maximum-likelihood bootstrap support followed by Bayesian posterior probability is shown at nodes, respectively. Genbank accession numbers follow species/genotype description. *Trypanosoma* species/genotypes isolated in Australia are in bold. Scale bar represents substitution per site.

Eight ZOTUs from 14 hosts including 10 woylies, 3 brushtail possums, and one chuditch (Table 1) grouped with the putative new species *Trypanosoma* sp. ANU2 (Fig. 1) that did not align with any known sequences. More detailed phylogenetic analysis revealed that *T*. sp. ANU2 is most closely related to *T. copemani,* although it appears to be a new species group (Fig. 3). The genetic distances between *T*. sp. ANU2 and *T. copemani* G1 were 9.7%, and between *T*. sp. ANU2 and *T. copemani* G2 were 10.3%.

### 3.2 Polyparasitism and diversity present in samples from different marsupial species

**Fig. 4:**
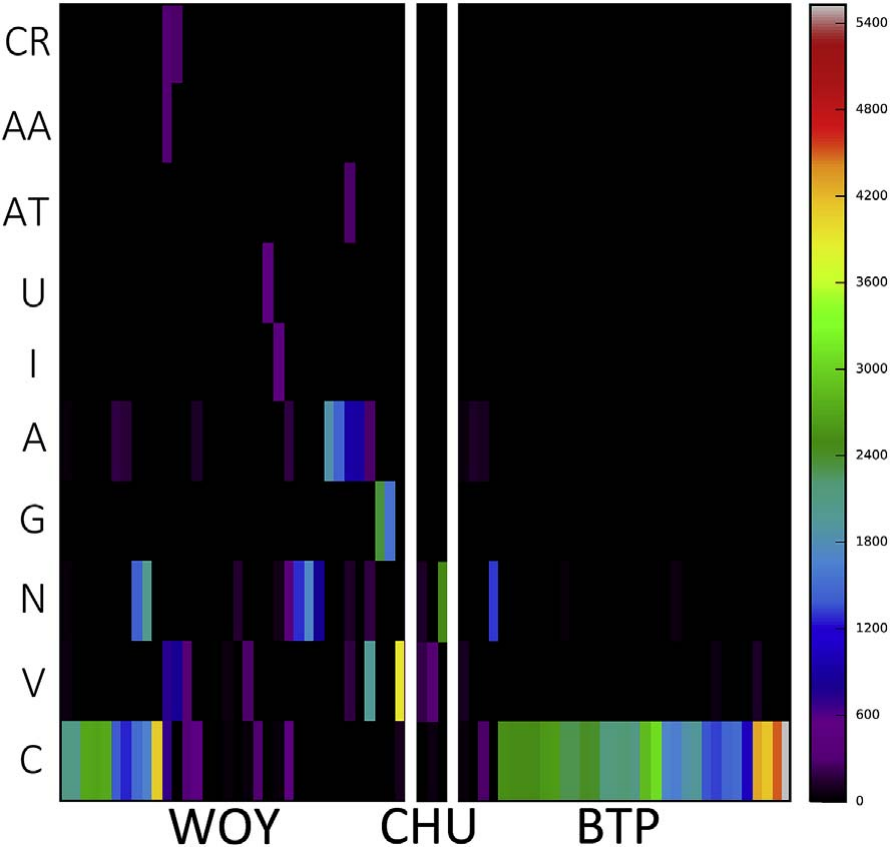
Abundance and diversity map of *Trypanosoma* spp. ZOTUs in different marsupial blood samples assigned to species groups shown in rows, while columns are individual blood samples. Samples are separated by host species including; WOY = woylie (34), CHU = chuditch (3), and BTP = brushtail possum (33). The colour scale indicates increasing number of sequences obtained from that sample, in that species, as shown in the intensity bar on the right. Species include; C = *Trypanosoma copemani*, V = *T. vegrandis*, N = *T. noyesi*, G = *T. gilletti*, A = *T.* sp. ANU2, I = *T. irwini*, AT = *T.* sp. AAT, U = unknown, AA = *T. avium*, and CR = *Crithidia* spp. (For interpretation of the references to colour in this figure legend, the reader is referred to the Web version of this article.)

**Fig. 5:**
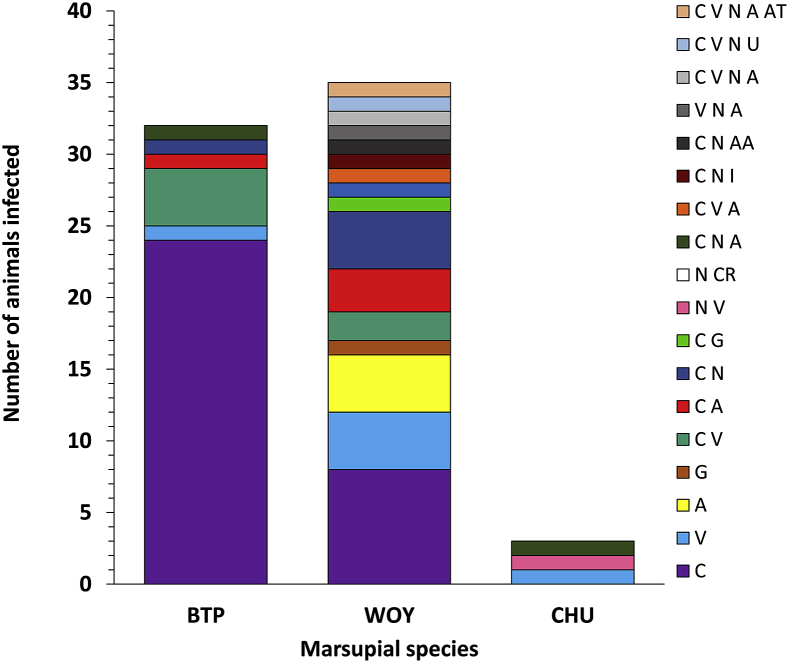
*Trypanosoma* spp. polyparasitism in 70 blood samples taken from marsupials in the Upper Warren Region. Marsupial species include: woylie (WOY), brushtail possum (BTP) and chuditch (CHU). *Trypanosoma* spp. include; C = *Trypanosoma copemani*, V = *T. vegrandis*, N = *T. noyesi*, G = *T. gilletti*, A = *T.* sp. ANU2, I = *T. irwini*, AT = *T.* sp. AAT, U = unknown, AA = *T. avium*, and CR = *Crithidia* spp.

**Fig. 6:**
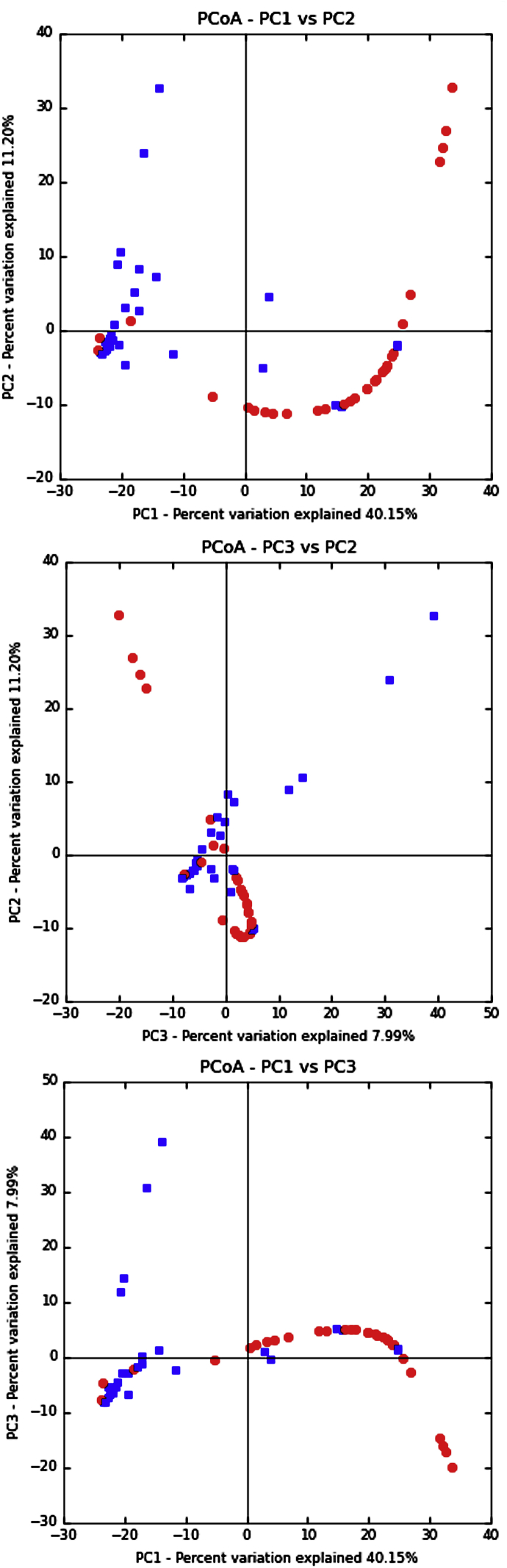
Principle Coordinates Analysis (PCoA) plots demonstrating relationship between *Trypanosoma* spp. ZOTUs and host species. Dissimilarity matrices were generated using sqrt transformed Bray-Curtis distances to show distance between abundance of ZOTUs between host marsupial species; the woylie (blue squares) and brushtail possum (red circles). (For interpretation of the references to colour in this figure legend, the reader is referred to the Web version of this article.)

Mixed infection with different trypanosome species and/or genotypes were identified in 35 samples from 35 hosts and was greater in woylies than in brushtail possums (Fig. 4, Fig. 5). Polyparasitism varied in number and some individuals had up to five different species assignments (Fig. 5). ZOTU diversity was greater in woylies than in brushtail possums, as measured by both species richness (for woylies S = 3.3 (95% CI 2.4–4.2), for brushtail possums S = 1.5 (1.1–1.9); z = 3.71, P = .0002) and the Shannon-Weiner index (for woylies H = 0.76 (95% CI 0.53–1.00), for brushtail possums H = 0.16 (0.03–0.29); z = 3.87, P = .0001). As well as differing in trypanosome diversity, host species also differed in ZOTU composition (Fig. 4). This difference was confirmed by ANOSIM (R = 0.24, p < .001) and the dissimilarity between abundance and diversity of ZOTUs between woylies and brushtail possums was visualised using principle coordinate plots (Fig. 6). SIMPER analysis showed that almost 80% of the dissimilarity between host species was due to differences in abundance of just seven ZOTUs, with ZOTU 1 (G1) being relatively more abundant in brushtail possums (Fig. 4), and ZOTUs 2 (G2), 3 (*T. noyesi*), 4 (*T*. sp. ANU2), 5, 7, and 85 (*T. vegrandis*) being relatively more abundant in woylies.

## 4 Discussion

This study is part of recent investigation into parasite diversity among woylies and other marsupials in the UWR of WA. Targeted amplicon NGS of the 18S rDNA loci in 70 blood samples from 56 wildlife hosts revealed: (1) extensive trypanosome diversity and polyparasitism, particularly among woylies; (2) a greater abundance of the potentially pathogenic G2 strain of *T. copemani* in woylies than in cohabiting brushtail possums; and (3) evidence that the current species-level taxonomy of Australian trypanosomes may need revision.

### 4.1 Parasite diversity

High within species diversity was observed with 85 ZOTUs being assigned in nine species groups, which cover the major trypanosome species identified in Australian wildlife including *T. noyesi*, *T. vegrandis,* and *T. copemani*. The 85 different ZOTUs that were identified presumably represent within-species diversity present between the different species of Australian *Trypanosoma*. This diversity should be treated with caution as the MiSeq platform has a small error rate (Quail et al., 2012), and errors often occur during PCR. However, error was accounted for by using UNOISE2 to filter and group sequences into ZOTUs in order to generate more biologically relevant sequencing data (Edgar, 2017). In addition to the unexpectedly high diversity found within *T. copemani*, discussed below, a large number of unique genotypes of *T. vegrandis* and *T. noyesi* were also isolated from woylies, brushtail possums, and chuditch. The large number of genetic variants could reflect the long period of isolation in Australia or suggest a wide range of vectors (Thompson and Thompson, 2015, Cooper et al., 2016). This high within-species diversity could also indicate genetic exchange, which occurs in a number of other trypanosomes such as *T. brucei* (Jenni et al., 1986), and *T. cruzi* (Gaunt et al., 2003), which forms hybrid species that increase genetic diversity.

ZOTU diversity was much greater among woylies than among brushtail possums. Brushtail possums were infected primarily with the G1 genotype of *T. copemani*. The woylie is known to host species-specific parasites (*e.g*. *Potoroxyuris keninupensis*, Hobbs and Elliot, 2016; *Ixodes woyliei*, Ash et al., 2017), but species specificity is not commonly seen in trypanosomes in Australia. Indeed, indigenous trypanosomes, especially *T. copemani* and *T. noyesi*, infect many different marsupials across the country (Cooper et al., 2016). There are a number of factors that could account for differences observed between the woylie and the brushtail possum. Ecological differences exist for example, the woylie is terrestrial while the brushtail possum is both arboreal and terrestrial (Wayne, 2005, Yeatman and Groom, 2012). Behavioural differences between the host species in their social interactions, foraging behaviours, and sheltering habits could influence the vectors that hosts are exposed to. Physiological differences such as stress and immune function could be contributing to more diverse parasite infections. Although, no real evidence exists that immune function is compromised in the remaining woylie populations, a collaborative study carried out in the UWR using faecal cortisol metabolite (FCM) concentration as an indicator of stress did find higher FCM concentrations in woylies (both translocated and resident) after translocation (Hing et al., 2017). Lastly, epidemiological factors such as population size and status between the species could also result in differences in their exposure to trypanosome vectors or their susceptibility to infection.

A number of interesting and unexpected findings were observed including the detection of avian trypanosomes in the woylie. This is not the first time that avian trypanosomes have been identified in marsupials in Australia with *T*. sp. AAT reported in the marsupial *Bettongia lesueur* (boodie) (Averis et al., 2009). The presence of avian trypanosomes in marsupials could be explained by opportunistic infection from increased susceptibility associated with depressed immune function and/or stress as discussed above (McInnes et al., 2011, Hing et al., 2017). *Trypanosoma irwini* was found for the first time in WA and this is the first occurrence of this parasite infecting a marsupial other than the koala (McInnes et al., 2009, McInnes et al., 2011, Barbosa et al., 2017). *Trypanosoma gilletti* was identified in the woylie and has been detected in the region before (Northover et al.; unpublished data). Although *T. gilletti* is closely related to *T. vegrandis*, as more genotypes of *T. vegrandis* are identified, the boundaries that separate these two species become increasingly unclear. Considering that the genetic relationships remain unclear and the morphology of *T. gilletti* has not been described, they could actually belong to the same species complex. However, further studies and longer DNA fragments would be required to explore this further.

Although a member of the trypanosomatidae family, the presence of *Crithidia* spp. in this study was unexpected considering they are known to be arthropod parasites. To our knowledge this is the first report of *Crithidia* spp. in marsupials. Woylies may be bitten by insects that carry chronic infections with *Crithidia* that would result in the amplification of these parasites in their blood even though they may not cause acute infection in the vertebrate host, similar to the avian trypanosomes. As with the avian trypanosomes, unusual trypanosome identifications in woylie blood may reflect facultative infections in an immunologically vulnerable host (Wayne et al., 2008, Hing et al., 2017).

### 4.2 Polyparasitism

Although it has been known for some time that marsupials in Australia host multiple *Trypanosoma* infections (Botero et al., 2013, Thompson et al., 2013a), it is only recently been reported that the composition of trypanosomes present in a single host can be detected by using NGS (Barbosa et al., 2017). Polyparasitism with trypanosomes was found to be extensive in the marsupials investigated here. Considering that *Trypanosoma* spp. infection in wildlife is presumed to be largely acute and under normal circumstances does not cause chronic infection or disease in the host (Hoare, 1972), this high level of polyparasitism is also unexpected. Multiple infections could result from frequent re-infection of the host from invertebrate vectors, or from the onset of chronic disease. However, it is difficult to understand parasite transmission or account for multiple infections with different trypanosomes without knowledge of the vectors that transmit them, or the capacity of Australian trypanosomes to cause disease. In *T. cruzi*, the causative agent of Chagas disease, polyparasitism is known to have an impact on disease progression and response to treatment (Perez et al., 2014), however, what happens when multiple trypanosome infections occur with Australian trypanosomes remains unknown.

Previous investigations into polyparasitism in the woylie looking at only *T. copemani, T. vegrandis*, and *T. noyesi* infections suggested that *T. vegrandis* may moderate sequential infection against *T. copemani* (Thompson et al., 2013b), although there is no known mechanism for this. It was also observed that woylies that became infected with *T. copemani* remained infected with *T. copemani* over time (Thompson et al., 2013b). However, whether this is due to constant reinfection or chronic disease cannot be accounted for in this study. It would be interesting to determine if these animals could maintain infection over time without constant exposure to vectors. It is likely that the prevalence of the three most common trypanosomes (*T. copemani, T. vegrandis,* and *T. noyesi*) may have been misrepresented in previous studies due to the limitations of only using the species-specific PCR primers or Sanger sequencing. Considering the high level of species diversity observed here, it is unlikely that species specific primers detect all genotypes present in these species groups. The recent improvement in molecular techniques in the last decade have made it easier to determine and investigate the true degree of polyparasitism and the impact this has upon the hosts in the future.

### 4.3 Trypanosoma copemani

Seventeen different ZOTUs were assigned to the species *T. copemani*. This was unexpected, as previous studies of *T. copemani* variants in woylies from a number of different populations had identified only G1 or G2 (Botero et al., 2013, Thompson et al., 2013b). This suggests that the true intraspecific genetic diversity could have been overlooked by traditional Sanger sequencing. The 17 ZOTUs found here, however, were separated into two separate clades, which aligned with published G1 and G2 sequences. The most abundant genotype in the dataset was almost identical to G1, although it differed by two indels compared to the published version (Botero et al., 2013) and was most common in brushtail possums. ZOTUs from the G2 clade were found more commonly in woylies, although a larger sample size would be necessary to understand the true prevalence of G2 in brushtail possums in the area. It is possible that G2 is transmitted by a vector that woylies are exposed to more often, thus accounting for the higher number of infections of G2 in the woylie (Botero et al., 2013, Thompson et al., 2013b).

This is the first time G2 has been detected in the brushtail possum, although G2 was previously isolated in the quokka (*Setonix brachyurus*), western quoll (*Dasyurus geoffroii*), quenda (*Isoodon obesulus*) (Botero et al., 2013), and more recently the koala (Barbosa et al., 2017). It should also be noted that blood samples were collected over a period of time, which may significantly influence parasite prevalence (Thompson et al., 2013b). Additionally, we cannot rule out that investigating blood samples accounts for acute infection only, thus G2, which may infect tissues and establish chronic infection (Botero et al., 2013, Botero et al., 2016), could be under-represented in this study. Although there is no discerning histology that suggests G2 has the capacity to cause disease in marsupials (Botero et al., 2016, Cooper et al., 2016), it has been observed inside mammalian cells indicating *T. copemani* may have a facultative intracellular stage. A higher prevalence of G2 was found in the UWR in the past (Botero et al., 2013), which combined with the presence of *T. copemani* DNA detected in tissues by PCR and observations of amastigote-like parasites that appeared to be inside cells *in vitro* led to the hypothesis that they could be contributing to the decline of the woylie. The two strains (G1 and G2) may constitute two separate species, although further research needs to be conducted in order to truly determine this (see below).

### 4.4 Implications for species-level taxonomy

Under an evolutionary or general lineage concept of species, a species represents a single lineage of organisms with a common evolutionary trajectory, distinguishable from other such lineages (Lymbery and Thompson, 2012). Under this concept, delimiting species is a two-step process: firstly determining that the putative species forms a monophyletic group in a comprehensive phylogenetic reconstruction of the genus; and secondly inferring the potential for this group to maintain a cohesive evolutionary trajectory. The novel trypanosome *T*. sp. ANU2 was identified in all three marsupial hosts and at all three sampling locations in the UWR. However, this genotype has not been knowingly observed in blood smears from infected animals. Recently operational taxonomic units phylogenetically similar to *T*. sp. ANU2 were identified in very small numbers in koalas (Barbosa et al., 2017). Here, *T.* sp. ANU2 was only identified after Sanger sequencing using dye for GC rich sequences and increasing the denaturation step in the sequencing reaction to prevent losing signal due to a long repeat region of thymine (data not shown). It was not possible to amplify DNA from any other loci apart from 18S rDNA. Further research, sequencing multiple loci and examining morphological and biological characteristics, is required to determine whether *T*. sp. ANU2 requires formal designation as a distinct species.

## 5 Conclusions

NGS technologies are vastly superior at establishing diversity present in a community by making it easier to identify polyparasitism and estimate prevalence more accurately. Future studies should focus on trying to understand the relationships that occur between these parasites and their marsupial hosts to describe the impact of trypanosomes in Australia. In the future it is vital that the invertebrate vectors of Australian trypanosomes are identified so the factors that influence their transmission and diversity can be better understood. Additionally, species-level taxonomy in trypanosomes in Australia needs to be revisited to describe the increasing number of species/genotypes that are detected by molecular investigations, and to provide supportive morphological and biological data. Increased understanding of the life histories of these trypanosomes will help to understand the role they play in their environment. This is especially important in areas like the UWR that support many susceptible marsupials in order to make informed decisions in the future regarding the health of the ecosystem and the animals that inhabit it.
