# Supplementary material for: Next generation sequencing reveals widespread trypanosome diversity and polyparasitism in marsupials from Western Australia
Source: Int J Parasitol Parasites Wildl. 2018 Jan 28;7(1):58–67. doi: 10.1016/j.ijppaw.2018.01.005 (PMC6031965; doi:10.1016/j.ijppaw.2018.01.005)
Supplement: Supplementary Table 1 [file mmc1.docx]

Supplementary Table 1: *Trypanosoma* spp. reference database used for taxonomic assignment. Species or genotype name is included along with Genbank accession number and host species.

| **Species/Genotype** | **Accession no.** | **Host** |
| --- | --- | --- |
| *Trypanosoma copemani* G1 | KC753530 | Woylie (*Bettongia penicillata*) |
| *Trypanosoma copemani G2* | KC753531 | Woylie |
| *Trypanosoma copemani* | EU571234 | Quokka (*Setonix brachyurus*) |
| *Trypanosoma copemani JD-2008b isolate P63* | EU571233 | Quokka |
| *Trypanosoma copemani* | EU571232 | Gilbert’s potoroo (*Potorous gilbertii*) |
| *Trypanosoma copemani JD-2008a isolate P94* | EU571231 | Gilbert’s potoroo |
| *Trypanosoma copemani H26* | AJ009169 | Wombat (*Vombatus ursinus*) |
| *Trypanosoma copemani AAI* | AJ620559 | Wombat |
| *Trypanosoma copemani AAP* | AJ620558 | Wombat |
| *Trypanosoma ABF* | AJ620564 | Wallaby (*Wallabia bicolor*) |
| *Trypanosoma AAT* | AJ620557 | Currawong (*Streptera* spp.) |
| *Trypanosoma irwini* | FJ649479 | Koala (*Phascolarctos cinereus*) |
| *Trypanosoma* sp. ANU2 | MF459652 | Woylie |
| *Trypanosoma vegrandis G3* | KC753533 | Woylie |
| *Trypanosoma vegrandis G4* | KC753532 | Woylie |
| *Trypanosoma vegrandis G5* | KC753534 | Woylie |
| *Trypanosoma vegrandis G6* | KC753535 | Woylie |
| *Trypanosoma vegrandis G7* | KC753536 | Woylie |
| *Trypanosoma noyesi G8* | KC753537 | Woylie |
| *Trypanosoma sp. AP-2011a isolate 15* | JN315381 | Brushtail possum (*Trichosurus vulpecula*) |
| *Trypanosoma sp. AP-2011a isolate 1* | JN315382 | Brushtail possum |
| *Trypanosoma sp. AP-2011a isolate 64* | JN315383 | Brushtail possum |
| *Trypanosoma sp. AP-2011b isolate 27 clone 2* | JN315384 | Woylie |
| *Trypanosoma sp. AP-2011b isolate 27 clone 1* | JN315385 | Woylie |
| *Trypanosoma sp. AP-2011b isolate 27 clone 3* | JN315386 | Woylie |
| *Trypanosoma sp. AP-2011b isolate 28 clone 11* | JN315387 | Woylie |
| *Trypanosoma sp. AP-2011b isolate 28 clone 2* | JN315388 | Woylie |
| *Trypanosoma sp. AP-2011b isolate 28 clone 4* | JN315389 | Woylie |
| *Trypanosoma sp. AP-2011b isolate 28 clone 8* | JN315390 | Woylie |
| *Trypanosoma sp. AP-2011b isolate 4 clone 10* | JN315391 | Woylie |
| *Trypanosoma sp. AP-2011b isolate 4 clone 6* | JN315392 | Woylie |
| *Trypanosoma sp. AP-2011b isolate 4 clone 8* | JN315393 | Woylie |
| *Trypanosoma sp. AP-2011b isolate 27 clone 4* | JN315394 | Woylie |
| *Trypanosoma gilletti* | GU966589 | Koala |
| *Trypanosoma noyesi H25* | AJ009168 | Kangaroo (*Macropus giganteus*) |
| *Trypanosoma noyesi TF3382* | KX008320 | Tabanid fly |
| *Trypanosoma noyesi TF2170* | KX008319 | Tabanid fly |
| *Trypanosoma noyesi TF1362* | KX008318 | Tabanid fly |
| *Trypanosoma noyesi TF1261* | KX008317 | Tabanid fly |
| *Trypanosoma noyesi TF1160* | KX008316 | Tabanid fly |
| *Trypanosoma noyesi TF1059* | KX008315 | Tabanid fly |
| *Trypanosoma noyesi TF857* | KX008314 | Tabanid fly |
| *Trypanosoma noyesi TF212* | KX008313 | Tabanid fly |
| *Trypanosoma noyesi TF27* | KX008312 | Tabanid fly |
| *Trypanosoma noyesi* | KU3544263 | Woylie |
| *Trypanosoma noyesi* | KX361179 | Brushtail possum |
| *Trypanosoma noyesi* | KX361178 | Brushtail possum |
| *Trypanosoma vegrandis* | KX361180 | Northern brown bandicoot (*Isoodon macrourus*) |
| *Trypanosoma vegrandis* | KX361181 | Northern brown bandicoot |
| *Trypanosoma vegrandis* | KX361182 | Northern brown bandicoot |
| *Trypanosoma teixeirae* | KT907061 | Little red flying fox (*Pteropus scapulatus*) |
